# The role of socio-demographic determinants in the geo-spatial distribution of newly diagnosed HIV infections in small areas of Catalonia (Spain)

**DOI:** 10.1186/s12889-020-09603-7

**Published:** 2020-10-09

**Authors:** Cristina Agustí, Núria Font-Casaseca, Francesc Belvis, Mireia Julià, Núria Vives, Alexandra Montoliu, Juan M. Pericàs, Jordi Casabona, Joan Benach

**Affiliations:** 1 Centre d’Estudis Epidemiològics sobre les Infeccions de Transmissió Sexual i Sida de Catalunya (CEEISCAT), Agència de Salut Pública de Catalunya (ASPC), Generalitat de Catalunya, Edifici Muntanya, Ctra Can Ruti, Cami de les Escoles s/n, 08916 Badalona, Barcelona, Spain; 2 Centro de Investigación Biomédica en Red de Epidemiologia y Salud Pública CIBERESP, Madrid, Spain; 3 Fundació Institut d’Investigació Germans Trias i Pujol IGTP, Badalona, Barcelona, Spain; 4 Department of Geography, Universitat de Barcelona, Barcelona, Spain; 5 Department of Political and Social Sciences, Health Inequalities Research Group, Employment Conditions Knowledge Network (GREDS-EMCONET), Universitat Pompeu Fabra, Barcelona, Spain; 6 Johns Hopkins University-Universitat Pompeu Fabra Public Policy Center, Barcelona, Spain; 7 Unit of Infections and Cancer - Information and Interventions (UNIC - I&I), Cancer Epidemiology Research Program (CERP), Hospitalet de Llobregat, Barcelona, Spain; 8 Cancer Screening Unit, Catalan Institute of Oncology, Hospitalet de Llobregat, Barcelona, Spain; 9 Vall d’Hebron Institute for Research (VHIR), Barcelona, Spain; 10 Infectious Disease Department, Hospital Clínic de Barcelona, Barcelona, Spain; 11 Departament de Pediatria, d’Obstetrícia i Ginecologia i de Medicina Preventiva i de Salut Pública, Universidad Autónoma de Barcelona, Bellaterra, Cerdanyola del Vallès, Barcelona, Spain; 12 Grupo de Investigación Transdisciplinar sobre Transiciones Socioecológicas (GinTRANS2), Universidad Autónoma de Madrid, Madrid, Spain

**Keywords:** HIV, Small areas, Income inequality, Neighborhood environment

## Abstract

**Background:**

Spatial visualization of HIV surveillance data could improve the planning of programs to address the HIV epidemic. The objectives of the study were to describe the characteristics and the spatial distribution of newly diagnosed HIV infection in Catalonia and to identify factors associated with HIV infection rates.

**Methods:**

Surveillance data from the national registry were presented in the form of descriptive and ring maps and used to study the spatial distribution of new HIV diagnoses in Catalonia (2012–2016) and associated risk factors at the small area level (ABS, acronym for “basic health area” in Catalan). Incident cases were modeled using the following as predictors: type of municipality, prevalence of young men and migrant groups, GBMSM activity indicators, and other variables at the aggregated level.

**Results:**

New HIV diagnoses are heterogeneously distributed across Catalonia. The predictors that proved to be significantly associated with a higher rate of new HIV diagnoses were ABS located in the city of Barcelona (IRR, 2.520; *P* < 0.001), a higher proportion of men aged 15–44 years (IRR, 1.193; *P* = 0.003), a higher proportion of GBMSM (IRR, 1.230; *P* = 0.030), a higher proportion of men from Western Europe (IRR, 1.281; P = 0.003), a higher proportion of men from Latin America (IRR, 1.260; P = 0.003), and a higher number of gay locations (IRR, 2.665; *P* < 0.001). No association was observed between the HIV diagnosis rate and economic deprivation.

**Conclusions:**

Ring maps revealed substantial spatial associations for the rate of new HIV diagnoses. New HIV diagnoses are concentrated in ABS located in urban areas. Our results show that, in the case of HIV infection, the socioeconomic deprivation index on which the Catalan government bases its budget allocation policies among the ABS should not be the only criterion used.

## Background

The World Health Organization recommends that not only should health outcomes, but also their societal distribution and determinants, be taken into account in public health monitoring in order to inform policies, programs, and practices aimed at improving the health of the population and achieving health equity [1]. Knowledge of the spatial incidence of HIV and associated variables can be of interest for the allocation of resources and the design of health policies, as well as for generating theories concerning transmission pathways.

Factors associated with the incidence of HIV infection have proven to be diverse and distributed both worldwide and within countries and transmission groups. Previous studies have revealed statistically significant differences in the distribution of new diagnoses by demographic characteristics and urban setting, with HIV positivity rates being higher in urban areas [2]. On the other hand, rural residents at risk for or living with HIV infection face unique challenges [2, 3], namely, they are less likely to have been tested for HIV or have been tested in the past year than urban residents [4].

Population mobility is also a key driver of the HIV epidemic [5] and is linked to a higher incidence of infection [6]. Although attention in recent years has focused on migrants from high-prevalence countries with generalized epidemics, there is evidence that in Europe, specific migrant groups from other regions with more concentrated epidemics, such as gay, bisexual, and other men who have sex with men (GBMSM) from Latin America and Western Europe, are also highly vulnerable to HIV infection [7].

Individual-level adverse health outcomes may be influenced directly or indirectly by the environment where a person lives [8–10]. Gay neighborhoods have been associated with HIV infection [11, 12], although there is some debate over whether living in a gay neighborhood is a risk factor or protective factor. Tieu et al. [9] associated living in a gay neighborhood with safer sex behaviors such as more frequent condom use for receptive and insertive anal sex. This agrees with the fact that individuals who perceive a higher sense of community and neighborhood participation are more likely to report fewer risk behaviors [9]. In contrast, living in a gay neighborhood has been associated with the use of drugs to enhance sexual experiences [11, 13].

Studies performed in the United States of America have reported that increased poverty and inequality are associated with the diagnosis of HIV and other sexually transmitted infections [14–16]. In contrast, a study performed in the United Kingdom showed an increased incidence of viral hepatitis and sexually transmitted infections but not HIV infection in deprived areas in the north of England [17]. Buot et al. [18] reported that high income inequality, low income, high unemployment, and high poverty correlate positively with the incidence of HIV. However, the relative importance of these factors differed with the population at risk: rates of infection by heterosexual contact were more significantly associated with income inequality and poverty, whereas GBMSM-linked cases were not significantly associated with socioeconomic indicators [18].

Catalonia, in the northeast of Spain has one of the highest rates of new HIV diagnoses in the country. In 2017, a total of 578 new diagnoses were reported, that is, 8.1 per 100,000 persons [19], which is higher than the European Union median (6.4 cases per 100,000 population) [20]. Men account for 86% of cases. Half of the diagnoses reported were in GBMSM (54%). Of these, 47% were diagnosed in people born outside Spain. More than half (58%) of all new diagnoses were made in the city of Barcelona. The median age of the cases was 36 years. Of all new HIV diagnoses in 2017, 38% were aged 30–39 years, 29% were ≥ 40 years, and 33% were under 30 years [19]. In Spain, studies on the effect of the geographical and socioeconomic context in the distribution of new HIV diagnoses are lacking.

The objectives of this study were to describe the characteristics and the spatial distribution of new HIV diagnoses in Catalonia using ring maps and to identify factors that are potentially associated with HIV infection rates at the small area level.

## Methods

We performed a cross-sectional ecological study using the “basic health area” (ABS [acronym in Catalan]) as the geographical study unit. The ABS is the regional demarcation used by the Catalan Health Service to organize primary healthcare services. An ABS includes a reference territory of a primary health care team and its population, which comprises 5000–25,000 people. Those ABSs with fewer than 5000 people were merged with bordering ABSs in order to protect the patient anonymity. During the study period, the limits of the ABSs were sometimes modified (basically ABS splits); this problem was solved by maintaining the original division and aggregating the data. Thus, we obtained a fixed number of 355 ABSs.

### New diagnoses of HIV data

We retrieved data on new HIV diagnoses reported from 2012 to 2016 in the Catalan HIV Surveillance Registry. The Registry is a population-based information system of all persons residing in Catalonia at diagnosis of HIV infection since 2001. The Registry provides the postal code, sex, country of birth, age, and transmission mode.

We calculated five-year aggregated new HIV diagnoses for each ABS. Multiple-year counts provide more stable estimates of HIV diagnoses, particularly in ABSs with small populations. New diagnosis rates per 100,000 persons were calculated by dividing the incident case count by the five-year sum of the population assigned yearly to each ABS.

### Exposure variables

To reflect territorial differentiation in Catalonia the ABSs were classified into five categories based on the characteristics of the municipality where each ABS is located, namely, geographical location (Barcelona and its metropolitan area and rest of the territory), population (municipalities with less than 10,000 inhabitants, 10,000–20,000 and more than 20,000 inhabitants) and population density (inhabitants/km^2^ of the municipality or municipalities where the ABS is located). When the ABS included more than one municipality and there was a discrepancy, the municipality of superior rank was prioritized. The five categories were urban Barcelona (ABS in Barcelona city), urban metropolitan (ABS in municipalities included in the metropolitan area of Barcelona), urban county (ABS in municipalities of more than 20,000 inhabitants and outside the metropolitan area of Barcelona), semi-urban (ABS in municipalities with a population of 10,000 and 20,000 inhabitants and a density of more than 150 inhabitants/km2, and, rural (ABS in municipalities with fewer than 10,000 inhabitants or a density of less than 150 inhabitants/km^2^.

The percentage of men aged 15–44 years among the assigned ABS population (year 2016) and the percentage of men from Western Europe and Latin America (year 2014) at the ABS level were calculated to account for differences in the proportion of men in the most common age range of new HIV diagnoses and the influence of the migrant population, respectively. The percentage for GBMSM as the transmission mode among new HIV diagnoses was calculated by ABS. Given the very small counts in many ABSs, the Agresti-Coull binomial proportion estimation was used to obtain less extreme values; this approach also has the advantage of imputing a value for ABS with zero incidence counts [21]. Relative socioeconomic disadvantage across Catalonia was evaluated using a socioeconomic deprivation index built for the assignation of budgets to the primary healthcare teams in Catalonia [22]. This index is a composite measure based on five indicators extracted from the national health registry of 2015, as follows: percentage of manual workers, percentage of people with a low educational level, rate of premature mortality, rate of avoidable hospitalization, percentage of population exempt from pharmaceutical copayment, and percentage of population with an annual income lower than €18,000. All these predictor variables were acquired from the set of basic health and health care indicators at the ABS level provided by the Agency of Health Quality and Evaluation of Catalonia (AQuAS) and the Information System for the Development of Research in Primary Care (SIDIAP).

Finally, to identify the gay neighborhoods, all gay locations (eg, bars, discotheques, saunas, hotels, shops, sex clubs, and travel agencies) located in Catalonia were retrieved from the Spartacus Guide and Universo Gay (2018 editions) and later georeferenced to their corresponding ABS. A total of 239 gay locations were mapped.

### Spatial visualization methods

Multivariate ring maps based on Huang et al. and López-De-Fede et al. [15, 23] were constructed. A ring map is a map surrounded by a set of concentric, segmented rings. Each ring displays an additional data dimension that represents an attribute of a particular location. We built maps with a base map of Catalonia in the center showing the rates of new HIV diagnoses for each ABS surrounded by a set of histograms with the distribution of the mode of transmission of HIV, sex, and country of birth for ABSs with new HIV diagnoses rates > 10 per 100,000 population. The ring maps were developed using QGIS and Inkscape, both are and free open source.

### Statistical analysis

The univariate distribution of the new diagnoses of HIV infection, as well as its association with the predictor variables, was examined from both the statistical and geographical perspective using bivariate statistics.

A negative binomial regression model with the population at risk as an offset was adjusted to account for the overdispersion of new diagnoses of HIV infection. The exponentiated coefficients of the model is the incidence rate ratio (IRR) relative to the reference category. Quantitative variables were scaled to make the IRRs comparable.

The Moran I test was used to check for spatial autocorrelation of the residuals, and a second model including an autocovariate based on the residuals of the first one was fitted. The AIC, BIC, and McFadden’s pseudo R^2^ were calculated to test the quality of the model. All calculations were performed using R.

The study was approved by the Ethics Committee of Hospital Germans Trias i Pujol.

## Results

The characteristics of the new HIV diagnoses reported in Catalonia during the study period (2012–2016) are presented in Table 1.

**Table 1:** Characteristics of the new HIV diagnoses reported in Catalonia (Spain), 2012–2016. N: 3220

	n	%	Rate of New HIV Diagnoses^a^	95%CI
**Sex**				
Male	2810	87.28	6.93	(5.75–8.11)
Female	410	12.73	1.04	(0.90–1.18)
Country of birth				
Spain	1890	58.69	4.76	(4.23–5.29)
Other	1330	41.30	3.2	(2.42–3.98)
**Transmission mode**				
Heterosexual men	470	14.60	1.18	(1.04–1.32)
Heterosexual women	313	9.72	0.8	(0.68–0.92)
GBMSM	2009	62.39	4.88	(3.78–5.98)
PWID	122	3.79	3.69	(2.77–4.61)
Other	306	9.50	0.8	(0.68–0.92)
**Total**	3220	100	7.97	(6.72–9.22)

^a^per 100,000 persons

GBMSM: Gay, bisexual and other men who have sex with men

PWID: People who inject drugs

Other: Parenteral transmission other than PWID, mother to child transmission and unknown

**Fig. 1:**
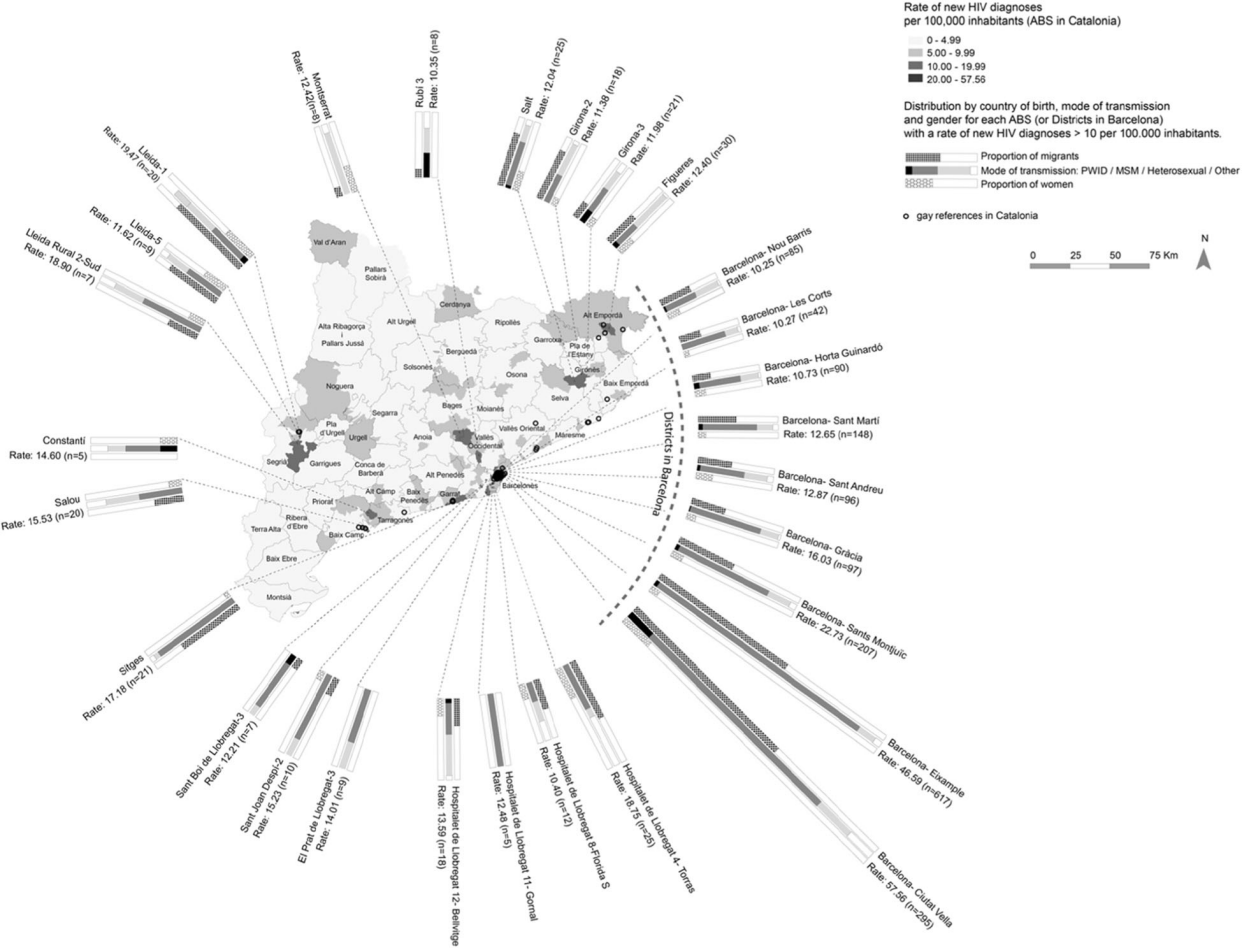
Rate of new HIV diagnoses per basic health area (ABS) in Catalonia (Spain), 2012–2016. N: 355

The choropleths in the base map of Fig. 1 show the rates of new HIV diagnoses. New HIV diagnoses are far from being homogenously distributed across Catalonia, with a gradient ranging from rural low-incidence areas to urban high-incidence areas and the highest rates in the ABSs of Barcelona city and its metropolitan area. A total of 22 ABSs out of 355 had no HIV diagnoses during the study period, while 31 had only one case.

A marked gender difference was observed in the diagnosis rate (Fig. 1), with men being the most affected by the HIV epidemic in Catalonia. The main mode of transmission of HIV was GBMSM in the vast majority of ABSs, with a diagnosis rate > 10 cases per 100,000 population. The percentage of new HIV diagnoses among the migrant population varied widely across Catalonia (Fig. 1).

**Fig. 2:**
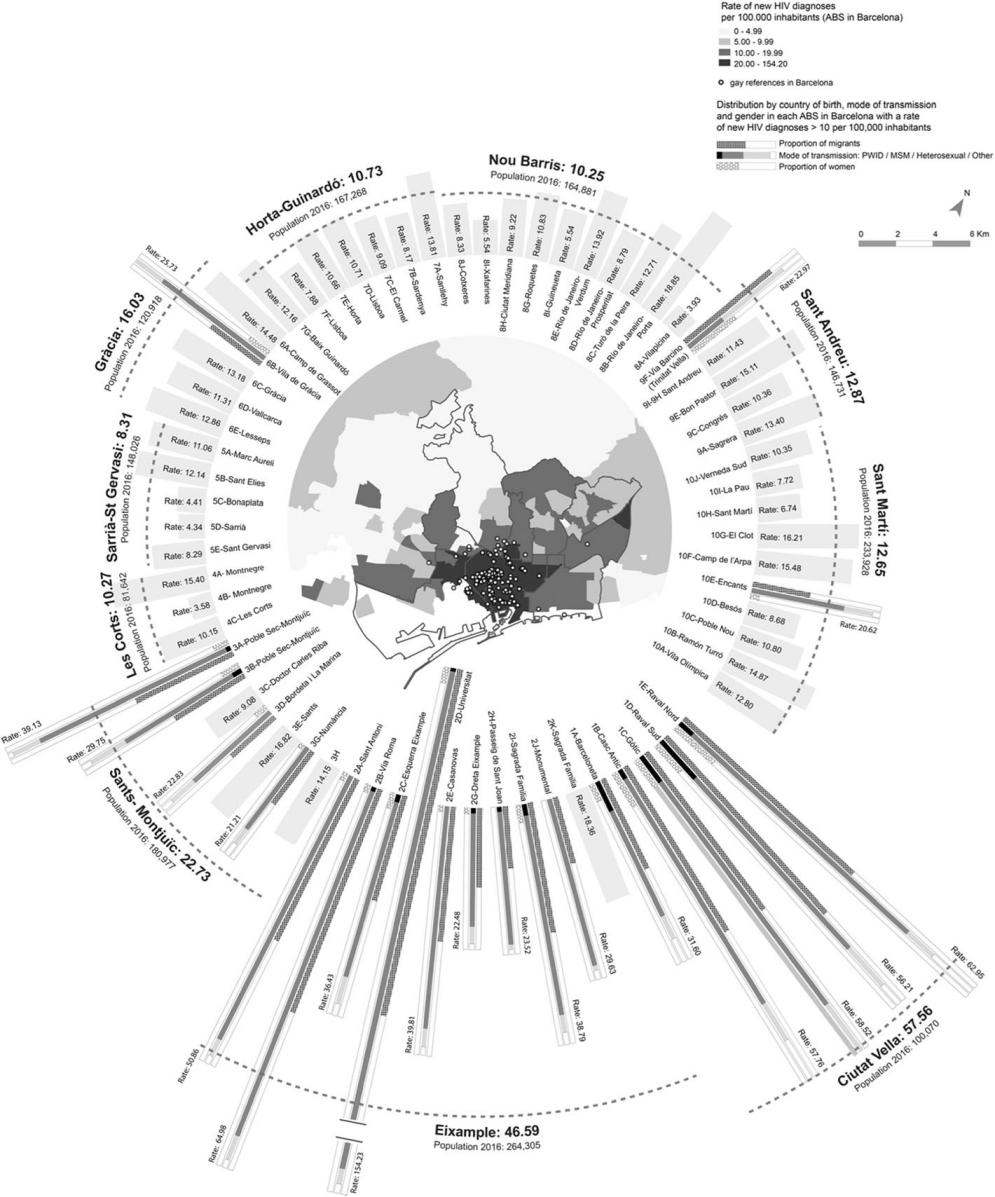
Rate of new HIV diagnoses per basic health area (ABS) in Barcelona and its metropolitan area, Catalonia (Spain), 2012–2016

The circles in the map of Figs. 1 and 2 show the gay locations. A zoom of Barcelona city and its metropolitan area is shown in Fig. 2. Both maps reveal a spatial association between the rate of new HIV diagnoses and the number of gay locations in the ABS. Gay locations were concentrated in downtown Barcelona city and in the village of Sitges. The ABS “Universitat” (Barcelona), which presented the highest rate, with 154.23 cases per 100,000 persons, was the ABS with most gay venues (*N* = 82) in Catalonia. The second ABS in number of gay references was Sitges (*N* = 40), with a new HIV diagnosis rate of 17.18 cases per 100,000 persons.

Mean new HIV diagnosis rates tended to increase in all cases, with the exception of the deprivation index, where the highest HIV diagnosis rate (11.6 cases per 100,000 persons) was found in the first quartile of less deprived ABS. The Pearson correlation with the diagnosis rate was positive and significant (*p* < 0.001) for all the factors considered except for the deprivation index (Table 2a). The mean new diagnosis rate was clearly higher for the city of Barcelona than for the rest of Catalonia (21.16 cases per 100,000 population), and type of municipality was significantly associated with HIV diagnosis rate (eta-squared = 0.289, p < 0.001) (Table 2b).

**Table 2:** Mean rate of new HIV diagnoses in Catalonia basic health areas (ABS) for each quartile (2a) / category (2b) of the risk factor, 2012–2016. N: 355

2a)	**Quartile**				**Corr.**^**b**^
	**1st**	**2nd**	**3rd**	**4th**	
Men, 15–44 years (%)	4.60	5.78	7.13	14.36	0.473***
Men, Western Europe (%)	5.01	5.49	5.71	15.64	0.491***
Men, Latin America (%)	3.90	4.74	6.70	16.52	0.318***
Gay references (count)^a^	6.22	30.50	50.14	67.44	0.725***
Infection from GBMSM (%)	5.31	4.77	5.93	17.68	0.410***
Deprivation index	11.60	6.56	5.29	8.40	−0.129*
2b)					
**Type of municipality**	**(n)**		**Corr.**^**c**^		
Rural	125	21.15	0.289***		
Semi-urban	89	7.97			
Urban (county)	26	3.66			
Urban (metropolitan)	48	5.31			
Urban (Barcelona)	67	7.03			
Total	355	6.21			

^a^Quantiles calculated at 95, 97.5 and 99.15% for this variable

^b^Pearson

^c^Eta-squared

^*^Significant at *p* < 0.05; **Significant at *p* < 0.01; ***Significant at p < 0.001

GBMSM: Gay, bisexual and other men who have sex with men

The statistical modeling of new HIV diagnoses in Model (1) showed significant spatial autocorrelation (Moran I, 0.163, p < 0.001, see Table 3), suggesting the need for correction; therefore, only Model (2) results (including the autocovariate) are commented on. The McFadden R^2^ value was 0.236, indicating adequate goodness of fit of the model. The ABSs that proved to be significantly associated with a higher rate of new HIV diagnoses were those located in the city of Barcelona (IRR, 2.520; *P* < 0.001), those with a higher proportion of men aged 15–44 years (IRR, 1.193; *P* = 0.003), those with higher proportion of MSM as the transmission mode (IRR, 1.230; *P* = 0.030), those with a higher proportion of men from Western Europe (IRR, 1.281; P = 0.003) and men from Latin America (IRR: 1.260 P = 0.003), and those with a higher number of gay locations (IRR, 2.665; P < 0.001). On the contrary, no association was observed between HIV diagnosis rate and economic deprivation (Table 3).

**Table 3:** Factors associated with the rate of new HIV diagnoses in basic health areas (ABS) of Catalonia (Spain), 2012–2016. N: 355

	MODEL (1)				MODEL (2)		
	Negative Binomial				Model (1) + Autocovariate		
	Coef.	(95%CI)		Pr(>|z|)	Coef.	(95%CI)	Pr(>|z|)
Variable (ABS level)							
Constant	0	(0–0)	0	< 0.001	0	(0–0)	< 0,001
Type of municipality							
Rural	Ref.				Ref.		
Semi-urban	1.147	(0.962–1.369)		0,126	1.118	(0.941–1.329)	0,204
Urban (county)	1.586	(1.231–2.043)		< 0.001 ***	1.44	(1.122–1.849)	0,004 **
Urban (metropolitan)	1.331	(1.063–1.669)		0,012 *	1.307	(1.048–1.630)	0,016 *
Urban (Barcelona)	2.52	(2.020–3.149)		< 0.001 ***	2.483	(2.004–3.080)	< 0.001 ***
Men, 15–44 years (%)	1.193	(1.113–1.280)		< 0.001 ***	1.189	(1.112–1.272)	< 0.001 ***
Men, Western Europe (%)^a^							
[0.02–2.13]	Ref.						
[2.13–13.2]	1.281	(1.085–1.513)		0,003 **	1.29	(1.098–1.516)	0,002 **
Men, Latin America (%)^a^							
[0.45–5.35]	Ref.						
[5.35–22.2]	1.26	(1.077–1.473)		0,003 **	1.246	(1.071–1.449)	0,004 **
Gay references (count)							
[0–2]	Ref.						
[3–6]	1.342	(0.995–1.824)		0,056	1.343	(1.009–1.799)	0,044
[7–82]	2.665	(1.924–3.742)		< 0.001 ***	2.529	(1.853–3.491)	< 0.001 ***
Infection from MSM (%)^b^							
[0.72–45.4]	Ref.				Ref.		
(45.4–55.6]	0.878	(0.733–1.052)		0,158	0.887	(0.744–1.057)	0,181
[55.6–67.1]	1.024	(0.865–1.212)		0,779	1.027	(0.871–1.210)	0,751
[67.1–95.2]	1.23	(1.016–1.488)		0,03 *	1.226	(1.019–1.475)	0,028 *
Deprivation index^b^							
[0.00–37.4]	Ref.				Ref.		
[37.4–46.4]	1.031	(0.859–1.237)		0,745	1.004	(0.842–1.198)	0,965
[46.4–54.5]	1.045	(0.865–1.264)		0,647	1.009	(0.838–1.214)	0,926
[54.5–100.0]	1.131	(0.923–1.387)		0,237	1.092	(0.896–1.332)	0,382
Autocovariate	–	–		–	1.156	(1.09–1.227)	< 0.001 ***
Model adjustment							
McFadden’s R2	0.226				0.236		
-2LL	− 1789.8				− 1767.4		
AIC	1823.8				1803.4		
BIC	1889.6				1873.1		
n	355				355		
g.l.	339				338		
Moran I test	0.163	0,033		< 0.001	−0.038	0,033	0,841

*p ≤ 0.05; **p ≤ 0.01; ***p ≤ 0.001

^a^1st-3rd vs. 4th quartile; ^b^quartiles

## Discussion

The main aim of this study was to identify socioeconomic risk factors associated with the rate of new diagnoses of HIV infection in Catalonia (Spain) and their spatial distribution. We performed an ecological study at the small area level based on surveillance data from the national registry of new diagnoses of HIV and publicly accessible data from the Agency of Health Quality and Evaluation of Catalonia and the Information System for the Development of Research in Primary Care. We also assessed the effect of the number of gay locations in the neighborhood.

Geographical information systems can help to better understand the spatial distribution of HIV and high-risk areas for disease transmission and acquisition [24, 25]. Our study showed the heterogeneous distribution of the rate of new diagnoses across Catalonia. Our data revealed differences for sex, transmission mode, and country of birth between small areas in Catalonia. The highest rates of new HIV diagnoses were in ABSs located in urban areas, especially the city of Barcelona. While differences between urban and rural areas have been reported [2], the fact that people living with HIV in rural areas are more likely to be diagnosed later than their urban counterparts [26, 27] should be taken into account. Rural residents at risk for or living with HIV experience barriers that include greater local stigma about HIV infection, a socially conservative climate, and scarcity of funding for widespread testing and treatment programs [3].

Our results indicate that a higher percentage of transmission via GBMSM in ABSs is associated with a higher incidence of HIV. This observation is consistent with the finding that GBMSM was the most common transmission mode in Catalonia during the study period, ranging from 53.7% of the new diagnoses of HIV infection reported among GBMSM in 2012 to 57.3% in 2016 [19]. We found that the HIV diagnosis rate was higher in ABSs with a higher number of gay locations. In Catalonia gay neighborhoods concentrate higher proportions of GBMSM than other areas of settings. Consequently, as GBMSM is the group most affected by the HIV epidemic in Catalonia, we can expect to find higher rates of new diagnoses in these neighborhoods. The probability of acquiring the infection by having HIV-positive people among one’s own sexual contacts is higher for people living in areas with a high prevalence of HIV. On the other hand, GBMSM living in gay neighborhoods often engage in the gay fast-lane subculture (defined as a way of life that is full of excitement, activity, and often risk), where drug use is frequently perceived to be normative [28]. Previous studies associated living in a gay neighborhood with the use of drugs to enhance sexual experiences and recent unprotected anal intercourse [11, 13]. Thus, the neighborhood itself could shape riskier sexual behavior through social networks and social norms.

The rate of new diagnoses of HIV infection is higher in ABS with a higher proportion of migrants from Western Europe and Latin America. Transmission of HIV has been severely affected by mobility. Migrants are considered a key group at risk for HIV infection [6], and the European Centre for Disease Prevention and Control (ECDC) and the European Commission consider them a priority group in HIV prevention and care [29]. In Catalonia, sex between men is the most frequent route of transmission among migrants from Western Europe (65%) and from Latin America (60.4%), who account for 19.8 and 70.5%, respectively, of all cases of HIV infection reported among migrant GBMSM [30] in Catalonia. The mode of transmission of HIV infection among migrants in Western Europe mimics epidemics in their countries of origin, that is, mainly through GBMSM. Conversely, transmission of HIV infection between men is less common in Latin America, and the high rates of HIV infection among GBMSM from Latin America may reflect selective migration of GBMSM from this region to Europe owing to the more permissive attitudes toward same sex relationships [7].

No association was observed between the socioeconomic deprivation index and the new HIV diagnosis rate. Other factors shown to be associated with the incidence of HIV infection among heterosexual men and women and people who inject drugs include high income inequality, low income, high unemployment, and high poverty, although these are not significantly associated with incidence among GBMSM [18]. Economic deprivation is not thought to be a significant factor, since the HIV epidemic in Catalonia is associated mainly with GBMSM. Previous studies have reported higher incomes among GBMSM residing in gay neighborhoods than in those who do not [31]. Generally, urban areas with a high significance for the gay community confirm the paradox of the post-Fordist city, i.e., ghettos develop in the city, although not all are marginal and some, for example, gay neighborhoods, show signs of urban elitization [32].

Our study has several limitations. Given that we report the results of an ecological study at the ABS level, the inferences drawn are only valid at this level and do not demonstrate any causal relationship. Furthermore, our results are based on the place of residence of newly diagnosed persons. However, place of residence and place of exposure may not necessarily be the same. Data on the rate of new diagnoses were obtained from the Catalan HIV Surveillance Registry, where 10–20% of cases are thought to be undiagnosed, as reported in similar registries [33]. The percentage of transmission among GBMSM is estimated from very small samples in many ABSs. In addition, no information was available on the mode of transmission in 9% of all diagnoses.

Nevertheless, it is important to remember that we used population-based sources of data on new HIV diagnoses and publicly available socioeconomic data. We complemented the results of the multivariate analysis to describe the distribution of new HIV diagnoses with geovisual representations using ring maps. Another strength of the study is the use of the ABS—the smallest administrative territorial health unit in Catalonia—as a geographical study unit. This approach could potentially facilitate future management of the HIV epidemic.

## Conclusions

Our results confirm the epidemiological pattern of the HIV epidemic observed in Catalonia. Nevertheless, the spatial analysis we performed provided us with a very accurate picture of the heterogeneous distribution of new diagnoses in Catalonia. We also determined factors related to ABS with a higher rate of new diagnoses. Our findings have important implications for the implementation of a scientific and public health response to HIV/AIDS. We showed that socioeconomic deprivation is not related to the incidence of HIV infection, thus indicating that the high rate of newly diagnosed HIV infection in certain small areas suggests that the socioeconomic level should not be the only criterion for budgetary allocation in the ABS. Furthermore, HIV prevention programs in Catalonia should continue to focus on GBMSM, mainly in urban areas. Our study identified geographical hotspots for HIV and could help guide the locations where HIV testing should be enhanced in primary care and where to implement specific HIV prevention campaigns.

## Data Availability

Data is publically available on demand by contacting the correspondence author: Cristina Agustí, cagusti@iconcologia.net.

## Abbreviations

ABS: Basic health area
AIDS: Acquired immune deficiency syndrome
GBMSM: Gay, bisexual and other men who have sex with men
HIV: Human immunodeficiency virus
IRR: Incidence rate ratios
PWID: People who inject drugs
